# LncRNA SNHG6 role in clinicopathological parameters in cancers

**DOI:** 10.1186/s40001-023-01358-2

**Published:** 2023-09-21

**Authors:** Khushbukhat Khan, Muhammad Irfan, Areej Abdul Sattar, Manal Bint Faiz, Anees ur Rahman, Hafsa Athar, Daniela Calina, Javad Sharifi-Rad, William C. Cho

**Affiliations:** 1 Department of Healthcare Biotechnology, Atta-ur-Rahman School of Applied Biosciences (ASAB), National University of Sciences and Technology (NUST), Islamabad, 44000 Pakistan; 2 Department of Clinical Pharmacy, University of Medicine and Pharmacy of Craiova, 200349 Craiova, Romania; 3 Facultad de Medicina, Universidad del Azuay, Cuenca, Ecuador; 4 Department of Clinical Oncology, Queen Elizabeth Hospital, Kowloon, Hong Kong

**Keywords:** LncRNA, SNHG6, Cancer, Oncogenes, Epigenetic control, Signal transduction, Neoplastic gene regulation

## Abstract

RNA sequencing has revealed that a substantial portion of the human genome undergoes transcription, yet a minimal fraction of these transcripts translates into proteins. LncRNAs, RNA molecules less than 200 nt in length, once deemed as transcriptional noise, have now emerged as crucial regulators of numerous cellular processes. This review focuses on the lncRNA SNHG6, aiming to elucidate its biogenesis, the pivotal roles it plays, and its mechanisms in facilitating the hallmarks of cancer. A comprehensive literature review and analysis were undertaken to delve into the biogenesis of SNHG6, its roles in cellular processes, and the mechanisms through which it contributes to the hallmarks of cancer. SNHG6 is a notable lncRNA, observed to be overexpressed in various cancer types; its perturbation has been linked to tumor progression, emphasizing its significance in oncogenesis. This lncRNA contributes to a range of cellular aberrations, influencing transcriptional, post-transcriptional, and epigenetic processes of mRNA, ultimately driving cancerous transformations. LncRNA SNHG6 serves as a potential biomarker and therapeutic target due to its association with tumorigenesis. Understanding its mechanism and role in cancer can pave the way for novel diagnostic and therapeutic strategies.

## Introduction

Long non-coding RNAs are a group of endogenous transcripts of about 200 nucleotides long that are not translated into proteins. LncRNAs account for more than 70% of the total non-coding RNAs in the cell [1–4]. They are distributed in the nucleus, cytoplasm, and mitochondria, and can be found in both linear and circular forms [5–7]. The function of lncRNAs is dependent on their cellular localization. The lncRNAs that are present in the nucleus are associated with transcriptional regulation and post-transcriptional mRNA processing. However, the lncRNAs located in the cytoplasm modulate translation mainly by acting as competing endogenous RNA and sequestering specific miRNAs [8–10]. Originally, lncRNAs were considered transcriptional noise with no function, however a significant amount of research has now established the role of lncRNAs in different cellular processes such as metabolism, aging, reproduction, development, and differentiation using different mechanisms including epigenetic, transcriptional, and post-transcriptional regulation [11–16]. The abnormal expression of different lncRNAs has also been reported to be involved in oncogenesis, migration, and invasion, by acting as an oncogene or tumor suppressor gene [17–20]. The expression levels of lncRNA in cancer and normal cells are compared to establish an association between the lncRNA and certain cancer. A significant amount of such studies has shown the overexpression of lncRNAs in various cancers [21–24]. LncRNAs regulate the expression of genes involved in different cellular processes that contribute to tumorigenesis such as cell cycle regulation, immune response, survival, mobility, and pluripotency using different mechanisms [25]. Studies have shown that lncRNAs use an epigenetic, transcriptional, and post-transcriptional mechanism to act as oncogene or tumor suppressors [26]. One of the most important mechanisms of post-transcriptional gene regulation of lncRNA is by acting as ceRNA to sequester specific miRNAs and prevent their inhibition on their native protein-coding gene targets affecting different cellular processes including proliferation [23], invasion [27], apoptosis [28], and metastasis [29]. Small nucleolar RNA host gene 6 (SNHG6) also referred to as U87HG is a lncRNA that belongs to the 5′TOP family and lies on chromosome 8q13.1 [30]. The association of SNHG6 in cancer was first studied by Chang et al*.* Their findings showed that the upregulation of SNHG6 induced epithelial-to-mesenchymal transition (EMT) and promoted tumorigenesis and metastasis in hepatocellular carcinoma (HCC) [31]. The overexpression of SNHG6 has been associated with the progression of tumor and poor prognosis in colorectal cancer [32], gastric cancer [33], prostate cancer [34], HCC [31] lung cancer [35], breast cancer [36], gliomas [37] and many more. lncRNA-SNHG6 promotes proliferation [37], migration [38], invasion [39], EMT [40], and metastasis [41] mainly by acting as ceRNA and sponging specific tumor suppressor miRNAs. Therefore, in the present study, the role of SNHG6 in modulating the cellular cascades and inducing carcinogenicity was discussed. Furthermore, the whole mechanism of SNHG6 in regulating miRNAs in promoting cancer hallmarks is also highlighted.

The primary objective of this review is to offer a consolidated understanding of the role of the long non-coding RNA SNHG6 in relation to various clinicopathological parameters observed in cancers; by examining existing literature, this review seeks to shed light on the biogenesis, regulatory mechanisms, and the overarching influence of SNHG6 in dictating the hallmarks of cancer. As the landscape of molecular oncology evolves, it becomes increasingly clear that lncRNAs play pivotal roles in tumorigenesis, progression, and response to treatment. SNHG6, in particular, has emerged as a molecule of interest, given its notable associations with multiple malignancies. A thorough review of its role serves several purposes: (i) diagnostic and prognostic value: understanding SNHG6’s interactions and influence can help in the early detection of cancers and might offer insights into prognostic outcomes; (ii) therapeutic implications: by understanding the mechanistic pathways influenced by SNHG6, potential therapeutic targets could be identified, paving the way for personalized treatments in oncology; bridging knowledge gaps: a comprehensive review helps in identifying areas that are under-researched, guiding future investigations in the realm of lncRNAs in cancer; educational value: for the broader scientific community, especially those not deeply entrenched in lncRNA research, this review serves as a resource to grasp the significance of SNHG6 in cancer biology; by delving into the intricacies of SNHG6 and its associations with cancer, this review underscores the molecule's importance and paves the way for further research and potential clinical applications.

## Review methodology

To provide an updated and comprehensive review on the role of LncRNA SNHG6 in clinicopathological parameters in cancers, a systematic search was undertaken across the following specialized databases: PubMed/MedLine, Scopus, Web of Science, Science Direct, TRIP database.

The objective of this search was to identify articles that specifically discuss the role and underlying mechanisms of SNHG6 in attaining the hallmarks of cancer. Studies were considered eligible if they: primarily focused on SNHG6's role in cancer biology; discussed the mechanisms by which SNHG6 influences cancer hallmarks; were available in full text. To ensure an exhaustive search and capture all relevant literature, the following Medical Subject Headings (MeSH) terms were used: “Cell Line, Tumor”, “Cell Movement”, “Cell Proliferation”, “Down-Regulation/genetics”.

“Epithelial-Mesenchymal Transition”, “Gene Expression Regulation, Neoplastic”, “Humans”, “MicroRNAs”, “Neoplasm Invasiveness/genetics”, “RNA, Long Noncoding”, “RNA, Long Noncoding/metabolism”, “Up-Regulation/genetics”. After the retrieval of articles based on the aforementioned criteria, they were screened for relevance and the most pertinent ones were selected for detailed review and the most representative data, findings, and mechanisms of action concerning SNHG6 were extracted. This information was then synthesized and summarized, with key insights being illustrated in tables and figures for enhanced clarity and understanding.

## SNHG6 biogenesis

RNA biogenesis is a multi-step, intricate process that takes place in the nucleus. Long non-coding RNAs (lncRNAs) represent a heterogeneous group of non-coding RNAs that are typically longer than 200 nucleotides. LncRNAs are the types of RNA with a 3′ polyadenylic tail and a 5′ methylated cytosine cap which are synthesized by RNA polymerase II enzyme. The biogenesis of lncRNAs is complex and varies depending on the type and location of origin in the DNA. While previously they were primarily associated with intergenic regions, it has become clear that lncRNAs can be transcribed from various regions of the genome [42]. The primary regions of DNA origin for lncRNAs: (i) long intergenic non-coding RNAs (lincRNAs) originate from the regions between genes; these regions were once thought to be "junk DNA", but are now recognized as areas that encode many lncRNAs with vital regulatory roles [43, 44]. From the region between two genes, i.e., intergenic region [43, 44]. LncRNA biogenesis is dependent and influenced by specific cell types and stage-specific stimuli [45]. Their synthesis occurs from various DNA genome components, including intergenic regions, promoters, and enhancers [46]; (ii) intronic regions: some lncRNAs are derived entirely from introns of protein-coding genes. These intronic lncRNAs can influence the expression of their host gene or other nearby genes [42]; (iii) exonic regions: these lncRNAs overlap with exons of protein-coding genes. They can be in the sense (same direction as the gene) or antisense (opposite direction) orientation; (iv) certain lncRNAs are transcribed from promoter regions (promoter-associated lncRNAs) or enhancer regions (enhancer RNAs or eRNAs); they often play roles in modulating gene expression by influencing the chromatin state; (v) some lncRNAs are transcribed from regions close to the transcription start sites of protein-coding genes but in the opposite direction, leading to bidirectional transcription [42]; (vi) lncRNAs can be transcribed in a way that they overlap entirely or partially with protein-coding genes; this can be in the same (sense) or opposite (antisense) direction [42].

**Fig. 1:**
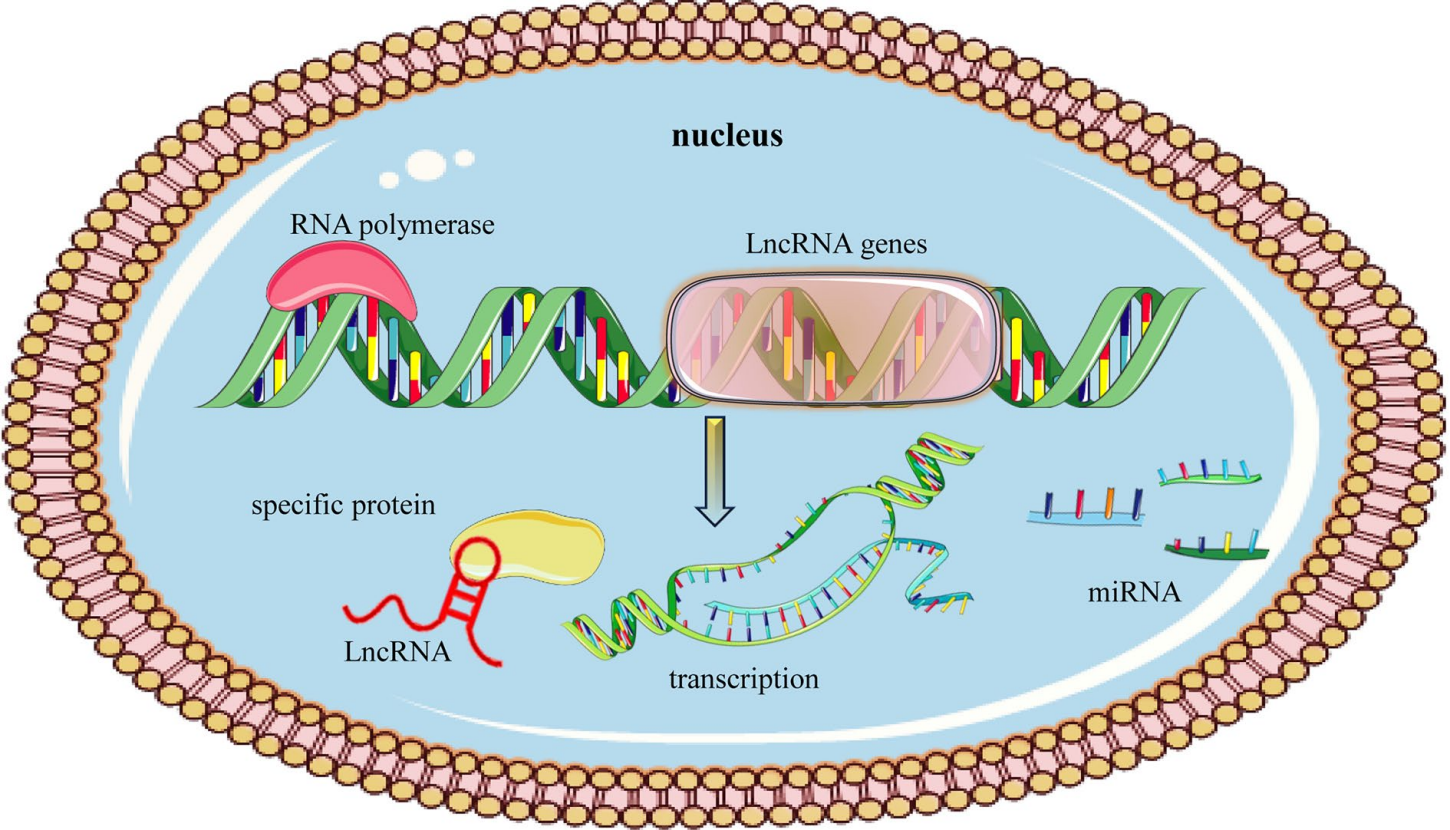
Biogenesis of LncRNA. This figure illustrates the multi-step process of long non-coding RNA (LncRNA) biogenesis; starting from the genomic DNA, the process is initiated with the transcription of LncRNA genes by RNA polymerase, similar to protein-coding genes. Once transcribed, these primary LncRNA transcripts undergo various modifications: LncRNAs receive a 5' cap which helps in stabilization and prevention of degradation; intronic regions are removed, and exons are joined together; not all LncRNAs are spliced, and some exist as single exonic molecules. A poly-A tail is added at the 3' end of the RNA molecule, some LncRNAs are non-polyadenylated and hence lack this tail; while many LncRNAs function within the nucleus, some are exported to the cytoplasm, employing mechanisms similar to mRNAs; depending on their sequence and structure, LncRNAs interact with various cellular molecules such as DNA, RNA, or proteins to exert their functional roles

LncRNAs can also be classified into nucleolar, cytoplasmic, and mitochondrial subtypes based on their subcellular location [47]. Referred to as small nucleolar RNA host genes, most snoRNAs are transcribed in the introns of protein and non-protein coding genes [48]. LncRNA known as small nucleolar RNA host gene 6 (SNHG6) was just recently found. SNHG6 is the housekeeping gene of the 5′TOP family and is found on chromosome 8q13 (Fig. 1). It can encode two different types of non-coding RNAs: SNHG6 RNA, which is synthesized by exons, and the other, U87 C/D box snoRNA which is generated by the second intron [9].

## SNHG6 role in cancer hallmarks

A tumor cell manipulates its microenvironment as well as the signal transduction to achieve the cancer hallmarks SNHG6 has been reported in many cancers which manipulated the downstream signaling and have been involved in autophagy, angiogenesis, cell proliferation, apoptosis, and metabolic modifications. These factors help the cells to metastasize and grow uncontrollably into tumor.

### Autophagy

**Fig. 2:**
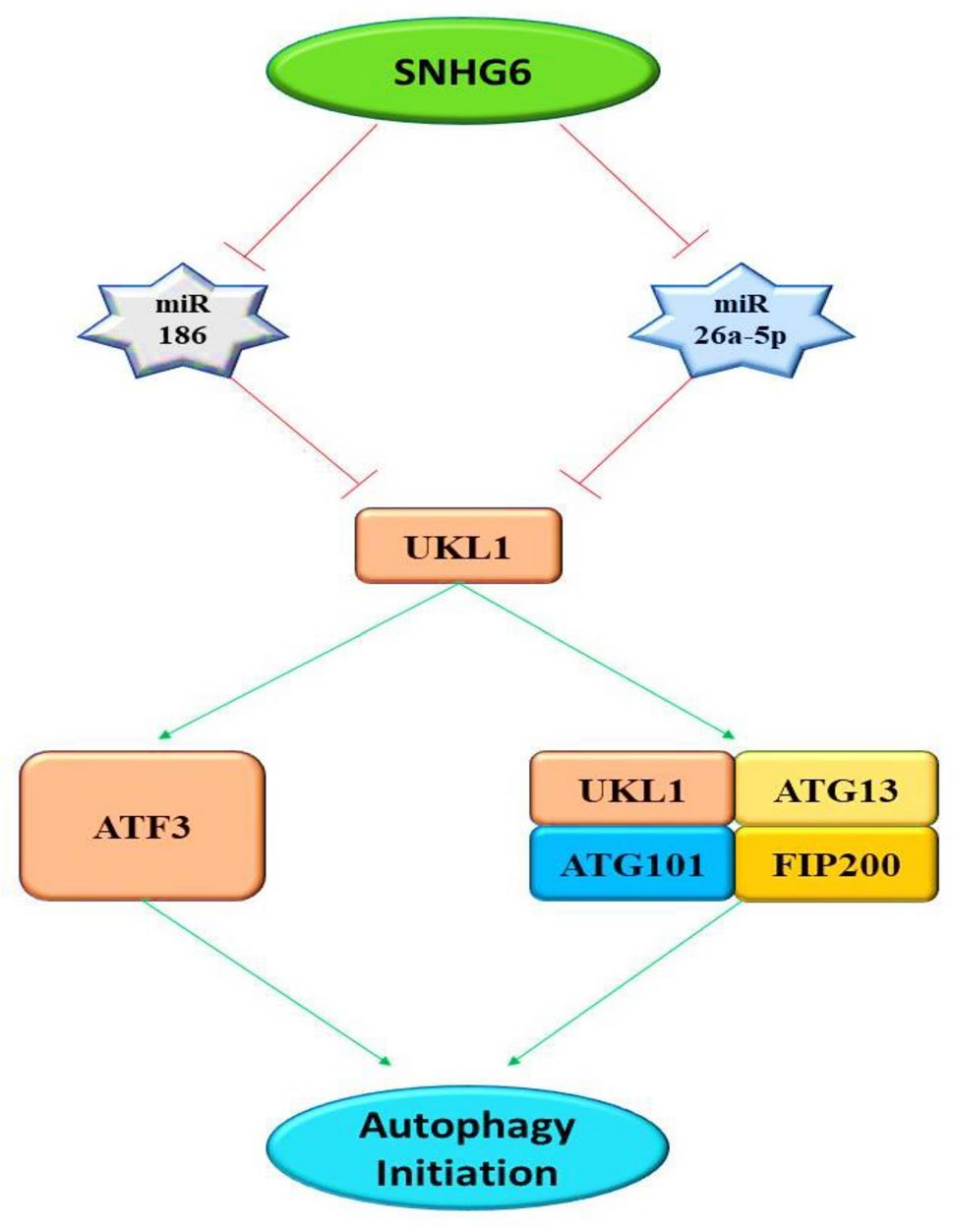
SNHG6 mediated regulation of autophagy. Autophagy can be initiated by the activation of the UKL1 complex. The regulatory RNAs, i.e., miR-26a-5p and miR186 act as an antagonist and inhibit the activations of the UKL1 complex thus shutting down the autophagy. SNHG6 inhibits these regulatory miRNAs and makes UKL1 available to a cell which leads to the ATF3 and UKL1 complex activation and autophagosome formation that helps in cell survival. Activating transcription factor 3 (ATF3); autophagy-related protein 13 (ATG13); autophagy-related protein 101 (ATG101); microRNA-186 (miR-186); small nucleolar RNA host gene 6 (SNHG6); unc-51-like kinase 1 (ULK1); focal adhesion kinase family interacting protein of 200 kDa (FIP200)

Often referred to as type 2 programmed cell death, autophagy is a process by which cells generally clear out their damaged components including protein, macromolecules, organelles, and sometimes pathogens. Cells use enzymes present in lysozyme to hydrolyze these components and the products are often reused in cellular processes [49]. Autophagy is mainly classified into three major types depending on the targets and mechanisms involved: (i) macro-autophagy is the most common type present in the cell. It includes the formation of a separate double-membranous organelle named an autophagosome that contains the target cellular debris and later on merges with the lysosome for hydrolysis of its contents that need to be cleared out; (ii) micro-autophagy does not involve any separate vesicle it directly involves the engulfment of targets by lysosomes from the cytoplasm; (iii) chaperone-mediated autophagy is the third type that is mostly dedicated to the damaged proteins. In this process, the autophagy is mediated by the chaperones that identify specific motifs in damaged proteins and lead them to lysosomal hydrolysis [50–52]. The fact that the process of autophagy clears out multiple cellular components as well as produce multiple products afterwards, makes it one of the most dynamic processes of cell that influences many physiological as well as pathophysiological pathways of the human body like cancer or infections [53–56]. Eukaryotic autophagy is regulated by multiple pathways and influenced by multiple factors inside and outside of the cell. On the molecular level, the UKL1 gene is considered to have a key role in the initiation of autophagy. AMPK, ATK, and mTOR genes are also gaining prime importance in the latest studies of molecular autophagy regulation. Starvation and stress-like conditions are the contributing factors that can activate the autophagy pathways [51, 57–60]. Long non-coding RNAs (lncRNAs) play significant roles in regulating various cellular processes, including autophagy. Specifically, the Small Nucleolar RNA Host Gene 6 (SNHG6) lncRNA is involved in regulating autophagy. It either directly or indirectly influences the process by modulating key mediators such as Unc-51-Like Kinase 1 (ULK1), Activating Transcription Factor 3 (ATF3), and Autophagy-Related Protein 13 (ATG13). [61–63] (Fig. 2). As autophagy plays important role in many physiological and pathophysiological pathways, the regulatory role of SNHG6 towards autophagy also provides it with the regulatory role in such processes that include autoimmune diseases, cancer progression, cancer inhibition, and chemoresistance [53, 61, 63].

Increased expression of SNHG6 in cells is correlated with the poor prognosis of osteosarcoma patients. SNHG6 knockdown osteosarcoma cells show reduced proliferation, migration, and invasion indicating a strong relation between SNHG6 expression and tumorigenesis as well as metastasis. miR-26a-5p is a regulatory micro-RNA in the cell that inhibits the UKL1 which is the initiator of the autophagy pathway. SNHG6 inhibits miR-26a-5p and in turn activates the UKL1. UKL1 becomes available in active form to initiate autophagy by activating the UKL complex and ATF3. The SNHG6-mediated autophagy induces the pro-tumor effects in osteosarcoma that are further confirmed by silencing the SNHG6 and disrupting the miR-26a-5p/UKL1 pathways showing anti-tumor effects in osteosarcoma cells [53, 62]. SNHG6-mediated autophagy can also induce chemoresistance in colorectal cancer cells against 5-fluorouracil, which is an approved drug against CRC. SNHG6 acts on miR-26a-5p and blocks it in CRC cells. The function of miR-26a-5p is to inhibit UKL1 in turn inhibiting the autophagy initiation. SNHG6 inhibits the miR-26a-5p that promotes UKL1 to initiate autophagy and reduce the 5FU-mediated apoptosis. SNHG6 promoted autophagy in turn leads to chemoresistance against the 5FU in the colorectal cells [61, 62]. Similarly in prostate cancer cells, SNGH6 inhibits miR-186 which leads to autophagy and ultimately causes chemoresistance against the paclitaxel drug [64]. Autophagy can be considered a “double agent” in the case of cancers as in some cases it may show anti-tumor activity contrary to previous examples in gastric carcinoma autophagy and apoptosis act together as anti-tumor pathways to get rid of the tumor. In such cases, the SNHG6 acts on the PI3K/AKT/mTOR signaling pathway activating these molecules further and in turn inhibiting apoptosis as well as autophagy. This inhibition of cell death results in the survival of cancer cells and poor prognosis of patients. SNHG6 overexpression results in increased cancer survival and metastasis so it can be used as a diagnostic as well as a prognostic marker [63, 65].

### Metabolic rewiring

Cancer was considered a genetic disorder throughout history, and it is one of the most complex genetic disorders to be present [66, 67]. With the contribution of Otto Warburg on altered glycolysis in cancer cells, it became evident that the cancer cells have altered metabolism and with further studies into this matter, the altered metabolism became one of the major hallmarks of cancer [68–70]. Nowadays the altered cancer metabolism and role of oncometabolite in cancer pathogenesis is a widely studied topic that includes the altered metabolism of glucose, amino acids, and other metabolisms. As Wishart once indicated, cancer is an extremely complex genetic disorder but a rather simple metabolic disorder, so treating cancer like a metabolic disorder might be the way to its better understanding and cure [70, 71]. SNHG6 is one of the factors involved in altered metabolisms of cancer cells. SNHG6 has known oncogenic effects through a lot of mechanisms. One of the most recently discovered mechanisms of which is through altering the metabolism of the cells. A study on colorectal cancers indicated that CRC cells have a higher level of SNHG6 than normal cells. In vitro and in silico analysis of interactions indicated that SNHG6 directly interacts with hnRNPA1 and causes the alternate splicing of pyruvate Kinase (PKM). It increases the amount of PKM2 with respect to PKM1. PKM2 is known to increase aerobic glycolysis and provide favorable conditions for tumorigenesis. The results were confirmed by making knockdowns of SNHG6 that resulted in a normal PKM ratio. This study provided evidence of SNHG6 involvement in metabolism alteration and ultimately cancer development [71, 72].

### Angiogenesis

Blood vessels emerge from the existing vasculature through a process known as angiogenesis. Angiogenesis normally begins in the capillaries and is crucial for the development, sustenance, and spread of malignancies [73]. Malignant cells require oxygen and nutrients for survival and growth, necessitating their proximity to blood vessels for access to the circulatory system and efficient waste removal. Angiogenesis is influenced by both stimulatory and inhibitory molecules. Over a dozen protein species have been identified as angiogenic activators and inhibitors. The level of expression of these angiogenic factors determines the invasiveness of malignant cells [74]. Long non-coding RNAs (lncRNAs) are the result of the transcription of different regions of the genome and they can alter the transcriptional and post-transcriptional levels of gene expression [75]. They can control gene expression by sponging endogenous microRNA or by any specific pathway, and as a result, they play crucial roles in numerous physiological processes, including cell growth and development as well as a malignant role like role in the development and progression of cancer [76]. Uncontrolled SNHG6 expression enhances the process of angiogenesis, tumor migration, invasion, metastasis, epithelial–mesenchymal transition (EMT), and chemoresistance while interrupting the malignant cell cycle and diminishing apoptosis [77].

### Apoptosis, metastasis, selective growth and proliferation

**Fig. 3:**
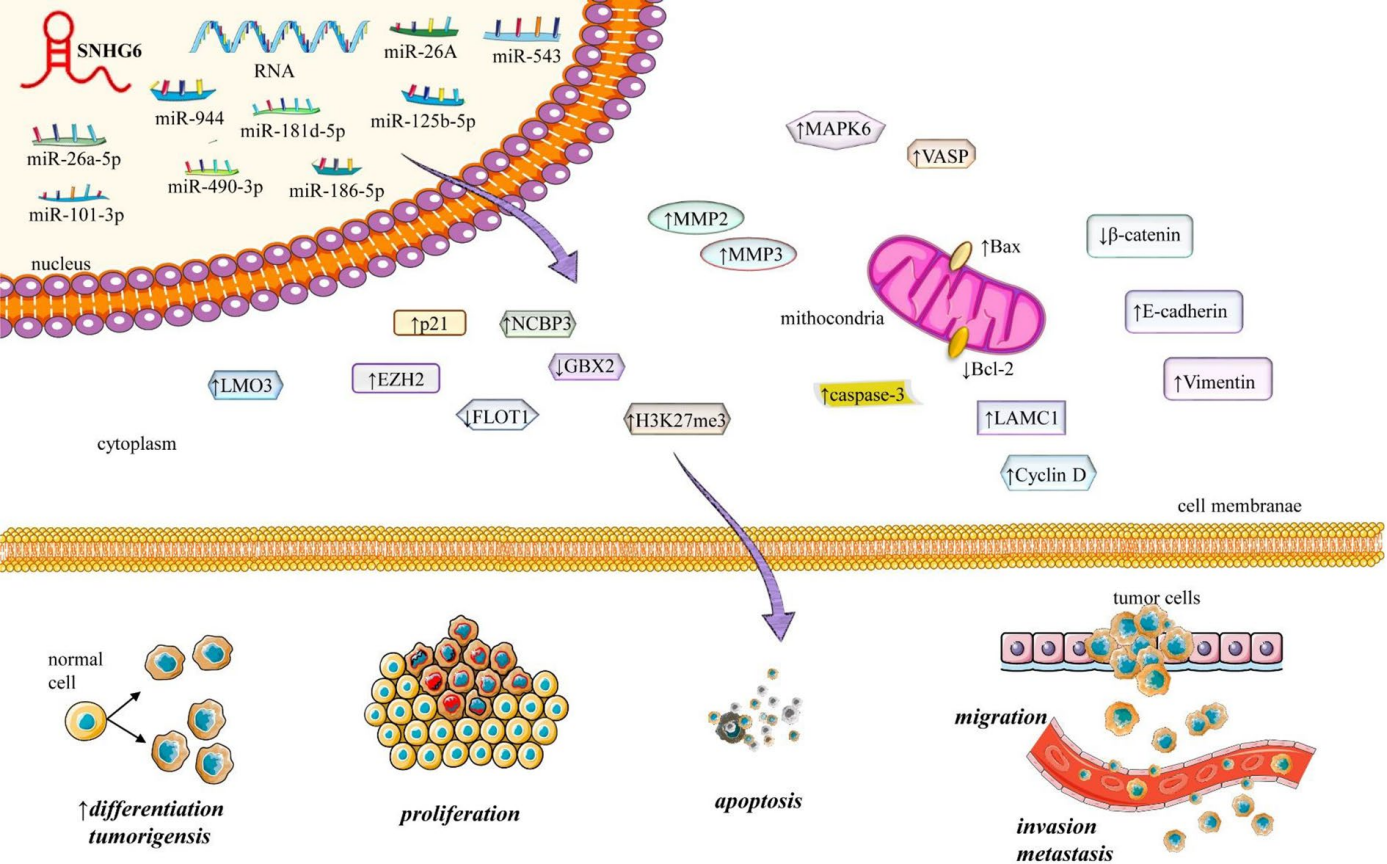
Diagram regarding the role of SNHG6 in the regulation of hallmarks of cancer. SNHG6 interacts with different miRNAs and activates the downstream signaling pathways;  this results in the perturbation in the signaling cascade resulting in uncontrolled cell differentiation, proliferation, tumorigenesis, inhibition of apoptosis, increase migration and metastasis. ↑increase, ↓decrease; enhancer of zeste homolog 2 (EZH2), nuclear cap-binding subunit 3 (NCBP3), LIM domain only 3 (LMO3), flotillin-1 (FLOT1), homeobox protein GBX-2 (GBX2), histone 3 lysine 27 trimethylation (H3K27me3), matrix metalloproteinases (MMP), mitogen-activated protein kinase 6 (MAPK6), vasodilator-stimulated phosphoprotein (VASP), laminin subunit gamma-1 (LAMC1), small nucleolar RNA host gene 6 (SNHG6)

SNHG6 predominantly acts as a ceRNA counteracting various tumor suppressor miRNAs. Essentially, SNHG6 "sponges" or pairs with complementary sequences in these tumor suppressor miRNAs [49]. When bound, the miRNA becomes incapacitated, unable to degrade, silence or hinder the translation of its downstream oncogenic genes. Consequently, this promotes cancer progression, impedes tumor cell apoptosis, and facilitates epithelial–mesenchymal transition (EMT). SNHG6 has been observed to exhibit increased expression in many cancer types [49]. SNHG6 has been reported to interact with miR-26a, miR-26b, miR-214/EZH2 axis [78, 79], miR-26a-5p/ULK1 axis [61], miR-6509/HIF1A [80], miR-760/FOXC1 axis [41], miR-1297/Bcl-2 axis [81], miR-429/FRS2 [82], miR-186-5p/HIF1α axis [83], miR-125b-5p/BMPR1B axis [84], and let-7c-5p/c-Myc axis [85], where it binds to complementary sequences in the miRNA, thereby releasing the mRNA suppressed by the miRNA. In the case of the miR-490-3p/RSF1 axis [35], SNHG6 sponges up miR-490-3p, releasing RSF1 from its bound which is responsible for regulating cyclin E1 [86], eventually promoting tumorigenesis. A number of reports have been made about SNHG6’s involvement with JNK pathway, p21, p27 [33, 37, 87, 88]. SNHG6 downregulates p21 and KLF2 while enhancing cyclin D1 activity [89] causing cancer cell proliferation and inhibiting apoptosis. By suppressing β-catenin and E-cadherin protein expression and promoting N-cadherin and vimentin translation SNHG6 not only increases cancer cell proliferation but also promotes EMT of cancer cells [90]. While in prostate cancer cells resistant to paclitaxel, increased SNHG6 expression leads to inhibition of miR-186 activity which is believed to be the cause of drug resistance and cell proliferation [64] (Fig. 3). Further investigation unveiled interlinking between SNHG6/miRNA/mRNA pathways. miR-543 sponging by SNHG6 leads to downstream expression of LAMC1 in breast cancer [91], while in glioma cells LMO3 gene is activated by binding of SNHG6 to miR-543 [92]. LAMC1 is a known regulator of the PI3K/AkT pathway and its suppression leads to the inhibition of cell proliferation and the Warburg effect in cancer cells [93]. Similarly, miR-485-3p/STYX axis in cervical cancer [94], and miR-485-3p/VPS45 axis in non-small cell lung cancer [95] are reported to be affected by SNHG6. By binding to miR-26a-5p, SNHG6 activates the ERK3/MAPK6 [36] and VASP pathway [96] from miR-26a-5p-induced repression. Similarly binding to miR-26b-5p affects the Hedgehog signaling pathway [97], and miR-15a sponging by SNHG6 has been seen to affect TAK1/JNK and Wnt/β-catenin signal pathways [98] promoting cancer cell growth, invasion, migration, EMT and represses tumor apoptosis. SNHG6 interacts with and inhibits UPF1 activity, activating the TGF-β/Smad pathway [31, 99]. miR-181 family is reported to be susceptible to SNHG6, and as a result, upregulation of JAK2 by SNHG6 overexpression has been reported to suppress apoptosis in Colorectal Cancer [100], where JAK2 is a known inhibitor of apoptosis and promoter of cancer growth [101]. One particular member, miR-181a-5p is reported to regulate cell cycle proliferation by targeting the E2F5 gene. Therefore, once miR-181a-5p is absorbed by SNHG6, E2F5 is activated, leading to cellular cell proliferation [102]. One of the most noted miRNAs is miR-101-3p [103]. It targets E2F8 [104], ZEB1 [31, 33], EZH2 [105], and more, all eventually controlling the proliferation, angiogenesis, EMT, and death of cells. The role of SNHG6 in cancer cell growth, metastasis, and apoptosis is indirectly regulated via its effect on the target miRNA and the following downstream pathway. SNHG6 binds to miR-944 and miR-181d-5p leading to an increase in ETS1 expression [106] eventually accelerating cancer cell proliferation, EMT, and inhibiting apoptosis. Conversely, in a study on colorectal cancer, SNHG6 expression was suppressed in tumor cells and as a result cell, proliferation and migration were elevated. Overexpressing SNHG6 resulted in the inhibition of ETS1 expression, thereby downregulating PI3K/AkT/mTOR expression and instigating cell death [107]. Therefore, the oncogenic or tumor-suppressing role of SNHG6 can only be determined by further investigation of its downstream pathways. Table 1 summarizes the multifaceted involvement of SNHG6 in different hallmarks of cancer.

**Table 1:** The role of SNHG6 in various cancer hallmarks

Cancer hallmarks	Mechanism of SNHG6 involvement	Clinical implications	References
Autophagy	↓UKL1, ↓ATF3, ↓ATG13 ↓miR-26a-5p, ↑UKL1 ↓miR-186 ↑Autophagy Modulate the PI3K/AKT/mTOR signaling pathway	Poor prognosis ↑Pro-tumor effects in osteosarcoma ↑Chemoresistance against 5FU in colorectal cancer cells ↑Chemoresistance against paclitaxel in prostate cancer cells Potential diagnostic/prognostic marker	[61–65]
Metabolic rewiring	directly interacts with hnRNPA1 ↑alternative splicing of PKM ↑PKM2, ↑PKM1 ↑ aerobic glycolysis	↑ Alteration metabolism in colorectal cancer cells ↑ Tumorigenesis Potential target for metabolic-based cancer therapies	[71, 72]
Angiogenesis	↑angiogenesis influences tumor migration, invasion, metastasis, EMT ↑chemoresistance	↑Development and spread of malignancies Alters malignant cell cycle, ↓apoptosis, allowing for cancer cell survival Potential target for anti-angiogenic cancer therapies	[73, 75, 76, 104]
Apoptosis, metastasis, selective growth, proliferation	↓various tumor suppressor miRNAs: ↓miR-26a, ↓miR-26b, ↓miR-214 regulates multiple signaling pathways: ERK3/MAPK6, VASP, JAK2, Hedgehog, via increasing miRNA sponging influences gene expression: ↑UPF1, ↑E2F5,↑ ETS1 ↑cell proliferation, ↑EMT ↓apoptosis ↑drug resistance by interacting with miRNAs (↑ miR-186 in prostate cancer cells)	Broad influence over multiple hallmarks of cancer ↑Cell growth, ↑Metastasis Potential as a therapeutic target, though further investigation is needed to understand its context-dependent roles Complexity suggests involvement in drug resistance and possible prognostic value	[49, 64, 78, 80, 83, 103, 106–108]

↑ increase, ↓decrease; SNHG6: small nucleolar RNA host gene 6; lncRNAs: long non-coding RNAs; CRC: colorectal cancer; hnRNPA1: heterogeneous nuclear ribonucleoprotein A1; PKM: pyruvate kinase M; PKM1: pyruvate kinase M1; PKM2: pyruvate kinase M2; EMT: epithelial–mesenchymal transition; miRNA: microRNA; ceRNA: competing endogenous RNA; EZH2: enhancer of zeste homolog 2; ULK1: Unc-51 like autophagy activating kinase 1; FOXC1: forkhead box C1; Bcl-2: B cell lymphoma 2; FRS2: fibroblast growth factor receptor substrate 2; HIF1α: hypoxia-inducible factor 1 alpha; BMPR1B: bone morphogenetic protein receptor type 1B; JNK: c-Jun N-terminal kinases; RSF1: remodeling and spacing factor 1; PI3K/AkT: phosphoinositide 3-kinases/protein kinase B; MAPK6: mitogen-activated protein kinase 6; VASP: vasodilator-stimulated phosphoprotein; TAK1: TGF-beta activated kinase 1; UPF1: up-frameshift suppressor 1; TGF-β: transforming growth factor beta; Smad: mothers against decapentaplegic homolog; JAK2: janus kinase 2; E2F5: E2F transcription factor 5; E2F8: E2F transcription factor 8; ZEB1: zinc finger E-box binding homeobox 1; ETS1: E26 transformation-specific sequence 1; mTOR: mammalian target of rapamycin; VPS45: vacuolar protein sorting 45

## SNHG6 as oncogene and ceRNA in cancers

LncRNAs serve regulatory roles in various molecular interactions within cells. Research has demonstrated the role of SNHG6 as a competing endogenous RNA (ceRNA) in multiple types of cancer. By acting as a sponge for tumor-suppressor microRNAs, SNHG6 prevents apoptosis and promotes epithelial-to-mesenchymal transition (EMT) characteristics. Experiments conducted in various cancer cell lines have shown that the oncogenic effects of SNHG6 can be reversed by artificially increasing the expression of its targeted microRNAs [85, 109]. Making it more important to know SNHG6 miRNA targets [49]. The overexpression of SNHG6 has been observed in many cancers, the increased expression is related to poor prognosis, tumor progression, and decreased survival rate. SNHG6 is involved in different tumor hallmarks including increased cell proliferation, evasion of apoptosis, metastasis, and invasion [49].

### Brain cancers

#### Glioma

SNHG6 association with poor prognosis and low survival is also reported in gliomas. Its elevated expression in glioma tissues and cell lines is responsible for cell proliferation, migration, and EMT [103]. To understand its contribution to malignancy, Cai and colleagues transfected normal astrocyte 1800 cells with pcDNA-SNHG6 and found the formation of a malignant phenotype. They further reported the involvement of p21-mediated cell proliferation behind the transformation from normal to malignant cells. To establish a relation between SNHG6 overexpression and p21 overexpression, they performed a loss of function analysis of SNHG6 and reported the attenuated p21 expression in SNHG6 silenced glioma U87 and 251 cells [37]. SNHG6 in vitro down-regulation caused a reduction in cell growth, proliferation, and migration while increasing apoptosis. In vivo inhibition of SNHG6 decreased tumor weight [37]. SNHG6 also sponges miR-101-3p in glioma to exert its oncogenic influence [103]. miR-101-3 targets ZEB1 in colon cancer and hepatic [31, 38] and chromodomain Y‐like (CDYL) in lung cancer [110]. But the target of miR-101-3p in glioma is not yet identified. High expression of SNHG6 has also been reported in gliomas, by other researchers [37, 111]. Li et al. demonstrated high expression of NCBP3 a nuclear cap-binding protein and SNHG6 in glioma cells. The NCBP3 increases the stability of SNHG6 through binding with its 5’-end. NCBP3/SNHG6 promotes tumorigenesis in glioma cells by inhibiting the transcription of GBX2 which is a tumor suppressor. The SNHG6 recruits PRC2 and binds with the promoter region of GBX2 to inhibit its expression by inducing H3K27me3 modification. The study further revealed that GBX2 is involved in decreasing the expression of FLOT1 an oncogene [112] (Table 2). The role of SNHG6 as an endogenous ceRNA in glioma was studied by Zhang et al. [113]. Using bioinformatics tools and wet lab experiments it was found that SNHG6 directly binds with miR-543 which has been previously identified as a tumor suppressor in ovarian [114] and breast cancer [115]. The study revealed that the knockdown of SNHG6 enhanced the expression levels of miR-543 while the elevated levels of SNHG6 reversed this effect confirming the direct relation of SNHG6 with miR-543 in glioma cells. Further experiments identified LMO3 as the target of miR-543, which has been previously found to be upregulated in HCC [116] and lung cancer [117]. A recent study [118] showed that SNHG6 promotes proliferation and EMT in glioma cells by acting as endogenous ceRNA and sponges miR-26a-5p. The miR-26a-5p targets PIM1 which is an oncogene involved in promoting proliferation and survival in gliomas [119, 120].

**Table 2:** Summary of research findings on the role of SNHG6 in various cancers

Cancer type	Function of SNHG6	Mechanism of action	Effect on cellular processes	Involved miRNAs	Affected genes and enzymes	References
Tongue and esophageal cancers	Promotes EMT, proliferation, and drug resistance	Down-regulates miR-186-5p, promotes HIF1α, interacts with EZH2	Increased metastasis, reduced apoptosis	miR-186-5p	HIF1α, EZH2, Bcl-2, MCL-1, Bax, caspase-3	[105, 130–134]
Gastric cancer	Inhibits apoptosis, promotes cell growth	Sequesters miR-101-3p, regulates EZH2	Increased metastasis, reduced apoptosis	miR-101-3p	EZH2, p21, ZEB1	[33, 81, 111, 135, 136]
Liver cancer (HCC)	Promotes tumor growth and metastasis	Interacts with UPF1, inhibits let-7c-5p, sponges miR-139-5p	Increased cell proliferation, reduced apoptosis	let-7c-5p, miR-139-5p	c-Myc, SERPINH1, TAK1, SETD7, LZTFL1	[31, 85, 138–140, 142, 143]
Pancreatic cancer	Counters drug resistance	Neutralizes miR-944	Improved drug sensitivity	miR-944	KPNA5	[144]
Colorectal cancer (CRC)	Promotes cell growth and metastasis	Regulates EZH2, inhibits p21, modulates miR-26a/b, miR-214	Increased metastasis, reduced apoptosis	miR-26a/b, miR-214	EZH2, E2F, p21, PKM, hnRNPA1	[38, 72, 79, 88, 102, 145, 146]
Tongue and esophageal cancers	Promotes EMT, proliferation, and drug resistance	Down-regulates miR-186-5p, promotes HIF1α, interacts with EZH2	Increased metastasis, reduced apoptosis	miR-186-5p	HIF1α, EZH2, Bcl-2, MCL-1, Bax, caspase-3	[105, 130, 132–134]
Gastric cancer	Inhibits apoptosis, promotes cell growth	Sequesters miR-101-3p, regulates EZH2	Increased metastasis, reduced apoptosis	miR-101-3p	EZH2, p21, ZEB1	[33, 81, 111, 135, 136]
Liver cancer	Promotes tumor growth and metastasis	Interacts with UPF1, inhibits let-7c-5p, sponges miR-139-5p	Increased cell proliferation, reduced apoptosis	let-7c-5p, miR-139-5p	c-Myc, SERPINH1, TAK1, SETD7, LZTFL1	[31, 85, 138–140, 142, 143]
Pancreatic cancer	Counters drug resistance	Neutralizes miR-944	Improved drug sensitivity	miR-944	KPNA5	[144]
Colorectal cancer	Promotes cell growth and metastasis	Regulates EZH2, inhibits p21, modulates miR-26a/b, miR-214	Increased metastasis, reduced apoptosis	miR-26a/b, miR-214	EZH2, E2F, p21, PKM, hnRNPA1	[38, 72, 79, 88, 102, 145, 146]
Bladder and kidney cancer	Oncogenic	Increases Snail1/2 for EMT; sequesters miR-125b	Increases cell proliferation, migration, invasion; induces apoptosis on knockdown	miR-125b, miR-15a	Snail1/2, NUAK1, p53, cyclin D	[98, 151]
Prostate cancer	Oncogenic; prognostic marker	Not fully elucidated; affects miR-186	Increases cell proliferation, invasion, migration; enhances PTX resistance	miR-186	Not fully elucidated	[34, 152]
Ovarian cancer	Oncogenic	Sponges miR-4465 and miR-543; regulates EZH2 and YAP1	Increases intravasation, metastasis, proliferation; induces EMT	miR-4465 miR-543	EZH2, YAP1	[153, 154]
Bone cancer	Oncogenic	Sponges miR-26a-5p; modulates KLF2, p21, ULK-1	Induces cell cycle arrest at G0/G1, growth inhibition, attenuates invasion; modulates autophagy and apoptosis	miR-26a-5p	KLF2, p21, ULK-1	[62, 89, 155]

EMT: epithelial–mesenchymal transition; miR-186-5p, miR-101-3p, let-7c-5p, miR-139-5p, miR-26a/b, miR-214, miR-125b, miR-15a, miR-4465, miR-543, miR-944: Types of microRNAs (miRNAs); HIF1α: hypoxia-inducible factor 1 alpha; EZH2: enhancer of zeste homolog; Bcl-2: B cell lymphoma 2; MCL-1: myeloid cell leukemia 1; Bax: Bcl-2-associated X protein; caspase-3: cysteine-dependent aspartate-directed protease 3; p21: cyclin-dependent kinase inhibitor 1A; ZEB1: zinc finger E-box binding homeobox 1; UPF1: up-frameshift suppressor 1; c-Myc: cellular myelocytomatosis viral oncogene; SERPINH1: serpin family H member 1; TAK1: transforming growth factor beta-activated kinase 1; SETD7: SET domain containing lysine methyltransferase 7; LZTFL1: leucine zipper transcription factor-Like 1; KPNA5: karyopherin subunit alpha 5; E2F: E2F transcription factors; PKM: pyruvate kinase M; hnRNPA1: heterogeneous nuclear ribonucleoprotein A1; Snail1/2: snail family transcriptional repressors 1 and 2; NUAK1: NUAK family kinase 1; p53: tumor protein p53; cyclin D: cyclin D protein family; PTX: paclitaxel; KLF2: Krüppel-like factor 2; ULK-1: unc-51 like autophagy activating kinase 1; YAP1: yes-associated protein 1; G0/G1: phases of the cell cycle

#### Pituitary tumors

In pituitary adenoma, SNHG6 expression dysregulation promotes adenoma cells’ viability, invasive potential, and EMT. Further, SNHG6-mediated silencing of miR-944 induces upregulation of RAB11A [121].

### Breast cancer

The carcinogenic role of SNHG6 is also explored in breast cancer. High expression of lncRNA SNHG6 in breast cancer tissues and cells has been reported to correlate with tumor size and metastasis [36]. Its elevated expression is also found in primary high-grade and progesterone receptor (PR) positive breast cancer, contributing to EMT and migration [122]. In vivo and in vitro knockdown of SNHG6 induced inhibition of cell proliferation, invasion, and metastasis [36]. siRNA-based silencing of SNHG6 in breast cancer MCF-7, SK-BR-3, and MDA-MB-231 cells led to growth arrest at the G1 phase and induction of senescence and apoptosis [39, 122]. SNHG6 serving as an endogenous sponge inhibits miR-26a and miR-26a-5p in breast cancer. Both miRNAs have tumor-suppressive roles in breast cancer and target MAPK6 and VASP at the post-transcriptional level. MiR-26a and miR-26a-5p suppression through SNHG6 promote proliferative, invasive, and migrational properties in breast cancer cells [36, 39]. Jafari Oliayi and colleagues [123] reported the importance of its splice variant SNHG6-203 as a prognostic marker and shed light on its application in breast cancer staging. According to them, SNHG6-203 expression is up-regulated in high-stage breast cancer than the low-stage. Also, its expression is upregulated in progesterone-negative breast tumors than progesterone positive. Despite their effort, more questions regarding SNHG6 differential expression in other breast cancer types: HER2 ±, Estrogen ±, and triple-negative rise. Studies demonstrating SNHG6 expression differences in all breast cancer types and also describing expression differences among each type could better facilitate understanding its role as a prognostic and diagnostic marker. SNHG6 promoted EMT and migration in breast cancer cells by sequestering miR-543 and hence elevating the expression of LAMC1, which is a target of miR-543. LAMC1 positively regulates the PI3K/AKT pathway. The increased levels of both SNHG6 and LAMC1 are associated with poor prognosis in breast cancer [124] (Table 2).

Another group demonstrated the role of SNHG6/miR-125b-5p/BMPR1B axis in triple-negative breast cancer. Elevated levels of SNHG6 were found in TNBC cell lines as compared to normal breast epithelial cells. Moreover silencing SNHG6 suppressed proliferation resulted in increased expression of miR-125b-5p in TNBC cell lines which further reduced BMPR1B expression. The overexpression of BMPR1B has been previously associated with enhanced migratory capabilities in TNBC through increasing CYP2J2 expression [125].

### Lung cancer

LncRNA SNHG6 up-regulated expression promotes malignant characteristics in non-small cell lung cancer (NSCLC) [7]. Li et al. 2020 demonstrated via Kaplan–Meier analysis that elevated SNHG6 expression is associated with poor survivability and high recurrence in lung adenocarcinomas (LAC) patients. They also reported that SNHG6 induces lymph node infiltration in NSCLC [110]. SNHG6 expression varies in different NSCLC cell lines. For example, its expression is reported higher in the NCI‐H460 cell line in comparison to A549 cells [110]. Overexpression of SNHG6 in the A549 cell line has a direct correlation with high protein concentrations of PCNA and MMP2 [7]. *In* vitro SNHG6 knockdown attenuated migration activity and proliferative vitality of A549 cells [7] while its ectopic expression restored cell viability and invasive properties [110]. In vivo knockdown of SNHG6 in BALB/c nude mice induced cell cycle arrest and caused a reduction in tumor weight and volume [110, 126]. In lung cancer, SNHG6 promotes ETS1 signaling by targeting its inhibitor microRNAs: miR-944 and miR-181d-5p. SNHG6 contains a binding site for these miRNAs and by recruiting Ago-2 it inhibits miR-944 and miR-181d-5p activity. ETS1 then induces the transcription of its target gene: WIPF-1, MMP-2 and MMP-3, and WIPF-1. These genes, in turn, bring about cell proliferation by stabilizing β-catenin and activating YAP/TAZ [7, 127] (Table 2). SNHG6 promotes cell cycle progression by inhibiting apoptosis via modulation of miR-490-3p expression [128]. SNHG6 induces poor survivability in NSCLC patients by sponging miR-101-3p and up-regulating the expression of CDYL [110]. In lung adenocarcinoma cells, SNHG6 sponges miR-26a-5p and regulate E2F7 expression leading to the attainment of cell motility and EMT properties [126]. The increased levels of SNHG6 were found to be associated with proliferation, migration, and invasion in NSCLC tissues and cells. The SNHG6 performs its oncogenic function by acting as ceRNA and sponging miR-485-3p the downstream target of which is the vacuolar protein sorting 45 homolog (VPS45) [95].

A recent study led by Li and colleagues explored the role of long non-coding RNA (lncRNA) small nucleolar RNA host gene 6 (SNHG6) in non-small cell lung cancer (NSCLC) [129]. The research employed multiple techniques such as fluorescence quantitative reverse transcription-polymerase chain reaction (qRT-PCR), colony formation assay, flow cytometry, MTT assay, and Western blotting to evaluate the expression levels and impacts of SNHG6 and p21 in NSCLC. The researchers found that SNHG6 expression was considerably elevated in NSCLC tissues when compared to normal tissues. In contrast, the expression of p21 was significantly lower in the NSCLC samples. A negative correlation was established between the expression of SNHG6 and p21; when SNHG6 was artificially silenced using small interfering RNA (siRNA) in specific NSCLC cell lines, a notable decrease in SNHG6 levels was observed, leading to a reduced cell viability; additionally, upregulation of SNHG6 was linked to increased cancer cell proliferation and the formation of malignant phenotypes. The study concluded that suppressing SNHG6 could inhibit cancer cell proliferation and encourage apoptosis in NSCLC cells; this anti-cancer effect was found to be mediated through the regulation of p21, a protein involved in cell cycle regulation and apoptosis. Overall, the findings suggest that SNHG6 could be a promising target for NSCLC treatment strategies [129].

### Digestive cancers

#### Tongue and esophageal cancers

In tongue cancer, high SNHG6 expression is responsible for EMT and aggressive cell proliferation [130]. SNHG6 expression is upregulated in esophageal squamous cell carcinoma (ESCC) and has an association with TNM stage, metastasis, and poor survival. Its knockdown in HET-1A and TE-1 cells reduced their proliferative and colony-forming capabilities and promoted apoptosis [131, 132]. Receiver operating characteristic curves (ROCs) indicated their significance as a diagnostic marker for predicting distant metastasis, lymph node metastasis, and TNM stage in ESCC [131]. SNHG6 employs its oncogenic role by downregulating miR-186-5p and promoting the co-expression of HIF1α [133] (Table 2). The upregulation of SNHG6 has been reported in esophageal squamous cell carcinoma [131]. The role of SNHG6 in regulating apoptosis in ESCC was demonstrated by Wang et al. [105]. Their findings demonstrated that the silencing of SNHG6 resulted in the promotion of apoptosis via decreasing the expression of anti-apoptotic genes like Bcl-2, and MCL-1 and elevating the levels of pro-apoptotic genes such as Bax and caspase-3.

The study by R. Tan et al. explores how the long non-coding RNA small nucleolar RNA host gene 6 (SNHG6) impacts the resistance of esophageal cancer (EC) cells to 5-fluorouracil (5-FU), a chemotherapy drug [134]. The study aims to understand the roles of SNHG6 and the enhancer of zeste homolog 2 (EZH2), specifically in this resistance and the findings revealed that SNHG6 was upregulated in EC cells; its presence facilitated cell colony formation and migration while inhibiting apoptosis, thus contributing to 5-FU resistance. When SNHG6 was silenced, the EC cell lines KYSE150 and KYSE450 showed increased sensitivity to 5-FU treatment, as observed through various cell viability and apoptosis assays. Further investigations into the underlying mechanisms pointed to SNHG6's role in modulating STAT3 and H3K27me3 levels through the promotion of EZH2. Elevated levels of EZH2, like SNHG6, increased the malignancy of EC cells and heightened their resistance to 5-FU. In experiments, the overexpression of EZH2 counteracted the beneficial effects of SNHG6 silencing on 5-FU sensitivity. The study elucidates that SNHG6 contributes to 5-FU resistance in EC cells by interacting with the EZH2/STAT pathway [134].

#### Gastric cancer

LncRNA SNHG6 dysregulation in gastric tumors, serum, and gastric cancer cell lines is reported to correlate with poor prognosis, TNM stage, lymph node, and distant metastasis [33, 135]. Its elevated expression has a direct correlation with the high expression of vitamin D receptors in gastric cancer tissues. Salehnezhad et al., demonstrated in a recent study that upregulated vitamin D receptor and SNHG6 have diagnostic power of 0.86 to differentiate malignant gastric tissue from non-malignant one [136]. The mechanism by which SNHG6 inhibits apoptosis was investigated and it was found that SNHG6 sequester miR-101-3p which has been previously identified as a tumor suppressor in several cancers such as gastric cancer [111], colorectal cancer, and cholangiocarcinoma [137]. The downstream target of miR101-3p was identified to be EZH2 (Table 2). SNHG6 suppression in gastric cancer cell lines halted cell growth and attenuated the migratory potential of cells. Two molecular events take place which reduces cancer cell viability: first JNK pathway activation and second EZH2 expression down-regulation which regulates the expression of senescence factor p21 [135]. SNHG6 employs EZH2 at the promoter region of p27 to induce its epigenetic silencing [33]. Also, it sponges miR-101-3p to promote ZEB1 expression [33] (Table 2). The role of the SNHG6/miR-1297/BCL-2 axis in regulating cisplatin resistance and progression of gastric cancer was studied. The levels of SNHG6 and Bcl-2 a known cell death inhibitor expression was found elevated in gastric cancer tissues in comparison to normal tissues. It was found that the upregulation of SNHG6 positively influenced B-2 expression by sequestering miR-1297 and the silencing of SNHG6 resulted in the repression of gastric cancer and DDP resistance [108].

#### Liver cancer

Numerous studies indicated the diagnostic significance of elevated lncRNA SNHG6 expression in hepatic cancer [138]. SNHG6 upregulation in hepatic cellular carcinoma (HCC) is reported to have an association with tumor growth and metastasis. It interacts with the UPF1 protein and induces cell proliferation by activating the TGF-β/Smad pathway. SNHG6 knockdown in xenografted mice led to a reduction in tumor volume. Silencing of SNHG6 halted cell progression and caused cellular death in hepatoma cell lines [31]. SNHG6 behaving as ceRNA in hepatocellular carcinoma exerts carcinogenic influence by modulating different miRNAs. For instance, it promotes cell proliferation by inhibiting let-7c-5p and inducing the expression of c-Myc and its downstream molecular targets [85]. miR-139-5p is also sponged by SNHG6 which is associated with high SERPINH1 levels and cell cycle acceleration in HCC cells [139]. SNHG6 causes EMT by suppressing miR-101-3p and positively modulating ZAB1 expression [31]. Its splice variant SNHG6-003 promotes drug resistance and short survival in HCC patients. SHNG6-003 carcinogenic influence pertains to its ability to complementary base pair with miR-26a/b and regulates transforming growth factor-β-activated kinase 1 (TAK1) expression [140].

LncRNA also can interact with different RNA binding proteins (RBPs) to form an RNA–protein complex which can then affect the stability of mRNAs or limit their access to translational machinery [141]. Wang et al. demonstrated how SNHG6 can promote tumor progression in HCC through a similar post-transcriptional mechanism. The study showed that silencing SNHG5 resulted in elevated levels of SETD7 and LZTFL1 mRNA in HCC cell lines. The RNA pull-down assay and RIP analysis identified HNRNPL and PTBP1 to be the protein binding partners of SNHG6. Both HNRNPL and PTBP showed binding sites for their target SETD7 and LZTFL1 mRNA, respectively. The study concluded that SNHG6 promotes HCC progression by recruiting HNRNPL and PTBP1 to post-transcriptionally inhibit SETD7 and LZTFL1 mRNA which then regulates EMT-related genes in HCC [142] (Table 2).

Kai Chen and colleagues [143] showed that increased expression of SNHG6 leads to elevated levels of Cyclin D1, Cyclin E1, and E2F genes which results in the promotion of tumorigenesis in HCC. Their findings demonstrated that overexpression of SNHG6 promotes the G1-S phase transition by binding to miR-204-5p and preventing its inhibitory action on E2F1.

Another recent study explores the role of long non-coding RNA SNHG6 in hepatocellular carcinoma (HCC) [85]. Researchers found that SNHG6 is highly expressed in various cancer types, particularly in HCC, and its high expression is linked to disease progression and poor patient outcomes. The study utilized gain and loss of function assays to demonstrate that SNHG6 contributes to the proliferation of HCC cells. Further analysis indicated a positive correlation between SNHG6 and the oncogene c-Myc, along with its downstream targets. The study revealed that SNHG6 functions as a competing endogenous RNA, absorbing microRNA let-7c-5p, thereby influencing c-Myc expression levels. This indicates that SNHG6 may play a key role in promoting HCC cell growth by interfering with the regulation of c-Myc [85].

#### Pancreatic cancer

A study conducted by Gao et al. focused on understanding the role of LncRNA SNHG6 in countering gemcitabine (GEM) resistance in pancreatic cancer [144]. GEM is a standard treatment for this type of cancer, but its efficacy is often hindered due to drug resistance. Researchers observed that lower levels of SNHG6 are linked with GEM-resistant pancreatic cancer and that higher SNHG6 levels are associated with better patient survival rates. Utilizing both bioinformatics and molecular techniques, the study reveals that SNHG6 can neutralize miR-944, leading to an increase in the expression of a target gene, KPNA5. Laboratory tests confirm that both SNHG6 and KPNA5 can inhibit the growth and spread of pancreatic cancer cells. More significantly, elevating the levels of these molecules made GEM-resistant cells more responsive to treatment. The researchers also note that KPNA5 is more prevalent in pancreatic cancer patients who have not developed GEM resistance. The study concludes that manipulating the SNHG6/miR-944/KPNA5 pathway could be a viable strategy for overcoming GEM resistance in pancreatic cancer, thereby potentially improving patient outcomes [144].

#### Colorectal cancer

Several studies demonstrated the elevated expression of lncRNA SNHG6 in different CRC cell lines and tissues by performing quantitative real-time PCR (qRT-PCR) [38, 88, 102]. Its high expression in CRC is partly because of DNA copy number gain and SP1 induction [145]. A strong association between SNHG6 upregulated expression and advanced tumor stages in CRC is reported [146]. Yu et al. further showed a positive correlation between high expression of SNHG6 and tumor size, advancement to TNM stage, and distant metastasis. Their findings were also backed by Wang et al. [38] who reported that SNHG6 high expression caused metastasis in RKO cells via the TGF-β/smad pathway and proliferation in HCT116 and RKO cells by up-regulating E2F. They also demonstrated via survival analysis that it is associated with poor survivability in patients having SNHG6 high expression [38]. In SW480 and HT29 cells, lncRNA SNHG6 brings about cellular proliferation by inhibiting p21 expression. It recruits EZH2 to the promoter region of p21 and causes H3K27me3 epigenetic modification hence, silencing the transcription of p21 [88]. SNHG6 suppression via miRNA brought about tumor proliferation inhibition, cell cycle progression arrest at the G0 phase, cell migration and invasion inhibition, and apoptosis [102]. SW480 and SW620 cells treatment with si-SNHG6 brought about a drastic reduction in the EMT properties of cells. Regression in the invasiveness of colon cancer cells is due to a decrease in the levels of N-cadherin, Snail, and Vimentin and an increase in E-cadherin levels [79]. A recent study reported SNHG6’s contribution to modulating aerobic metabolism in CRC. SNHG6 regulates the expression of pyruvate kinase M (PKM), an enzyme of the glycolysis pathway which converts phosphoenolpyruvate (PEP) to pyruvate [72]. Two isoforms of PKM are known: PKM1 and PKM2, generated through alternate splicing. The expression of PKM2 is reported higher in several cancers [147]. hnRNPA1 plays important role in PKM mRNA splicing and generation of PKM2 mRNA transcript. Mechanistically, SNHG6 induces hnRNPA1 to bind and splice PKM transcript in CRC cells, hence, sensitizing cancer cells for aerobic glycolysis sensitizing cancer cells for aerobic glycolysis [72]. LncRNA SNHG6 acts as ceRNA and upregulates EZH2 expression by suppressing its modulator miRNAs: miR-26a/b and miR-214 [79, 145]. Behaving as ceRNA, it also frees FOXC1 from modulatory control of miR-760 by binding and subsequently represses miR-760 [41]. SNHG6 enhances signal transduction via the wnt/β-catenin pathway by targeting miR-101-3p and promoting invasive capabilities in HT-29 and SW620 cells [111]. It also promotes the translation of the E2F5 mRNA transcript by sequestering miR-181a-5p [102]. SNHG6 was also found to be involved in negatively regulating the expression of miR-181 family members including miR-181a-5p, miR-181b-5p, miR-181c-5p, and miR-181d-5p in CRC. All four members of the miR-181 family were upregulated when SNHG6 was silenced, and this effect was reversed when SNHG6 was overexpressed. Further investigation of the SNHG6/mir-181 axis showed that the miR-181 family targets JAK2 by binding with its 3’-UTR [148]. SNHG6 causes chemoresistance by making an axis with microRNAs and modulating numerous factors. SNHG6 promotes CRC cell resistance to 5-fluorouracil (5-FU) by binding to miR-26a-5p and regulating ULK1. ULK-1 is responsible for initiating autophagy and enhances ULK-1-induced autophagy and is positively correlated with the inhibition of 5-FU-induced apoptosis and 5-FU resistance in CRC RKO cells [149] (Table 2). Recent evidence demonstrated the reversal of radioresistance in LoV and SW620 cells by the treatment of *Panax notoginseng* saponins (PNS). PNS repressed SNHG6 expression while simultaneously triggering the expression of miR-137 which being tumor-suppressor miRNA inhibits cell proliferation and induces apoptosis [150].

The study by Su Meng et al. delves into the role of long non-coding RNA small nucleolar RNA host gene 6 (SNHG6) in the progression of colorectal cancer (CRC), a leading cause of cancer-related mortality. The study explores how SNHG6 affects cell proliferation, invasion, and migration, focusing on its interaction with ETS1 (E26 transformation-specific sequence 1) and its role in the PI3K/AKT/mTOR signaling pathway. Interestingly, the study finds that SNHG6 is downregulated in colorectal cancer tissues, contrasting its upregulation in some other types of cancer. Conversely, ETS1 levels were found to be elevated in CRC tissues. Overexpressing SNHG6 in colon cancer cell lines had a significant inhibitory effect on cell proliferation, invasion, and migration. The study also reveals that this inhibition occurs through the targeting of ETS1 via the PI3K/AKT/mTOR pathway and this implies that SNHG6 might act as a tumor suppressor in the context of colorectal cancer, as opposed to its oncogenic role in other cancer types. The study suggests that SNHG6 could directly suppress ETS1, inhibiting the viability and proliferation of CRC cells [107].

### Bladder and kidney cancers

Wang and colleagues studied the elevated levels of SNHG6 in bladder cancer [151]. The study demonstrated that increased expression of SNHG6 causes EMT in bladder cancer via increasing Snail1/2. Their findings further showed that SNHG6 serves as ceRNA and sequesters miR-125b thus increasing the expression of kinase family 1 (NUAK1) (Table 2). SNHG6 overexpression is also reported as a cause of Wilms tumors or nephroblastoma, si-SNHG6 transfection of G401 and SK-NEP-1 cells by Su et al. revealed SNHG6 contribution in cell proliferation, migration, and intrusion. SNHG6 knockdown abolished cancer cells’ proliferative capabilities by downregulating p53 and cyclin D expressions and induced apoptosis. miR-15a was reported as a direct target of SNHG6 in Wilms tumor. SNHG6 inactivation positively modulated miR-15a expression which disrupted signaling via TAK1/JNK and Wnt/β-catenin pathways [98].

### Prostate cancer

Overexpression of lncRNA is also found in prostate cancer and Yan et al. demonstrated its significance as a prognostic marker [34]. Bioinformatics’ tools and GO and KEGG pathway analysis have been used to indicate its potential miRNA and mRNA targets and co-expressed genes in prostate cancer. But functional validation of these outcomes in wet lab experimentation is still required. Investigations on SNHG6 binding proteins also remain. Gain and loss of function analysis may prove useful in further understanding the crucial SNHG6 role in prostate carcinogenesis. The role of SNHG6 in the chemo-resistance of prostate cancer was determined by Cao et al. [152]. They found that levels of SNHG6 were highly increased while miR-186 was found to be downregulated in drug-resistant prostate cancer tissues. The silencing of SNHG6 not only decreased cell proliferation, invasion, and migration, but also elevated the sensitivity of prostate cancer tissues to paclitaxel (PTX) confirming the role of SNHG6 in PTX resistance (Table 2).

### Ovarian cancer

SNHG6 elevated expression, as determined by qRT-PCR, is associated with poor survival rate, enhanced intravasation, and distant metastasis in ovarian cancer. In vivo and in vitro Loss of function studies provided evidence that SNHG6 knockdown not only inhibits cancer cell metastasis but also its proliferation. MiR-4465 has sponged it in ovarian clear cell cancer which leads to enhanced EZH2 expression [153].

A study conducted by Su et al. investigates the role of the long non-coding RNA small nucleolar RNA host gene 6 (SNHG6) in the progression of ovarian cancer [154]. Specifically, the study aims to understand whether SNHG6 exerts its oncogenic functions via the miR-543/YAP1 pathway. The research found that both SNHG6 and Yes-associated protein 1 (YAP1) were significantly upregulated in ovarian cancer tissues compared to adjacent normal tissues, while the expression of miR-543, a known tumor suppressor, was substantially reduced. Experiments showed that overexpression of SNHG6 in ovarian cancer cell lines SKOV3 and A2780 significantly boosted cancer cell proliferation, migration, invasion, and EMT. On the contrary, SNHG6 knockdown had opposite effects on these cellular activities. The study also demonstrated a negative correlation between the levels of SNHG6 and miR-543 in ovarian cancer tissues. Overexpression of SNHG6 was shown to inhibit miR-543 expression, whereas its knockdown increased miR-543 levels. Furthermore, the oncogenic effects of SNHG6 were found to be counteracted by a miR-543 mimic and intensified by an anti-miR-543 agent. YAP1 was identified as a target of miR-543, and its overexpression was able to reverse the effects of SNHG6 down-regulation on the malignant behaviors of ovarian cancer cells. In conclusion, the study reveals that SNHG6 plays a role in promoting malignant phenotypes of ovarian cancer cells, and this action is mediated through the miR-543/YAP1 pathway [154].

### Bone cancer

In osteosarcoma, SNHG6 is associated with advanced stages of cancer and poor survival [89]. Loss of function of SNHG6 resulted in cell cycle arrest at G0/G1 phase, growth inhibition, attenuated invasion, and induced cellular death due to upregulation [62, 89]. It modulates the expression of KLF2 and p21 to perform its oncogenic function [89]. It also has a role in autophagy and apoptosis modulation. It inhibits ULK-1 at transcriptional level by sponging miR-26a-5p [62]. ULK-1 suppression prevents activation of its downstream targets ATF3 and caspase3 which allows the cells to escape apoptosis [155]. Table 2 provides a summary of the regulatory roles of SNHG6 in different types of cancers, focusing on its mechanism of action, the affected miRNA targets, the affected downstream genes, and the biological outcomes.

## Conclusion and future perspectives

LncRNA SNHG6 has been identified as a critical player in the pathogenesis and progression of several types of cancers, including breast, colon, and prostate cancer. It serves as an influential regulator in a variety of biological processes crucial to tumorigenesis, such as cell proliferation, apoptosis, migration, invasion, and EMT. Elevated levels of lncRNA SNHG6 have been linked to key clinicopathological parameters such as tumor size, lymph node invasion, and advanced tumor stage. This lncRNA is also involved in modulating cancer cells' sensitivity to chemotherapy and targeted therapies, making it a potential target for overcoming therapeutic resistance. lncRNA SNHG6 has been found to regulate the Wnt/β-catenin signaling pathway, commonly associated with cell proliferation and migration; it also affects the PI3K/Akt pathway which is crucial for cell survival and apoptosis; also lncRNA SNHG6 serves as a sponge for miR-26a, affecting downstream genes involved in EMT. Another interaction involves miR-101, affecting cancer cell apoptosis. Given these multiple roles across different signaling pathways and interacting ncRNAs, lncRNA SNHG6 holds significant potential as a biomarker for early cancer detection, risk assessment, and prognosis prediction. Therapeutic strategies targeting SNHG6 may offer new avenues for treatment, particularly in cancers characterized by high SNHG6 expression. To translate these promising in vitro and in vivo findings into clinical practice, the initiation of phase I/II clinical trials is recommended. In summary, lncRNA SNHG6 is not only a pivotal regulator in the cancer landscape, but also a prospective therapeutic target. As we continue to understand its mechanistic roles and interactions, the hope is that personalized therapeutic approaches can be developed, maximizing efficacy while minimizing side effects.

## Data Availability

Yes.

## Abbreviations

5-FU: 5-Fluorouracil
ATF3: Activating transcription factor 3
ATG101: Autophagy-related protein 101
ATG13: Autophagy-related protein 13
Bcl-2: B cell leukemia/lymphoma 2 protein
BMPR1B: Bone morphogenetic protein receptor type 1B
CDYL: Chromodomain Y-Like
ceRNA: Competing endogenous RNAs
c-myc: Cellular myelocytomatosis oncogene
CRC: Colorectal cancer
CYP2J2: Cytochrome P450 family 2 subfamily J member 2
E2F7: E2F transcription factor 7
EMT: Epithelial to mesenchymal transition
ERK: Extracellular signal-regulated kinase
ESCC: Esophageal squamous cell carcinoma
ETS1: ETS proto-oncogene 1, transcription factor
EZH2: Enhancer of zeste homolog 2
FIP200: Focal adhesion kinase family interacting protein of 200 kDa
FLOT1: Flotillin 1
GBX2: Homeobox protein GBX-2
GO and KEGG: Gene Ontology and Kyoto Encyclopedia of Genes and Genomes
H3K27me3: Lys-27 in histone 3
HCC: Hepatocellular carcinoma
HER2: Human epidermal growth factor receptor 2
HIF1α: Hypoxia inducible factor 1 subunit alpha
HNRNPA1: Heterogeneous nuclear ribonucleoprotein A1
JNK: C-Jun N-terminal kinases
KLF2: Kruppel-like factor 2
LAC: Lung adenocarcinomas
LAMC1: Laminin subunit gamma 1
LMO3: LIM domain only 3
lncRNAs: Long non-coding RNAs
LZTFL1: Leucine zipper transcription factor like 1
MAPK: Mitogen-activated protein kinase
MCL-1: Induced myeloid leukemia cell differentiation protein
miR: MicroRNA
MMP: Matrix-metalloproteinases
NCBP3: Nuclear cap binding subunit 3
NSCLC: Non-small cell lung cancer
NUAK1: NUAK family kinase 1
pcDNA: Plasmid cloning DNA
PCNA: Proliferating cell nuclear antigen
PEP: Phosphoenolpyruvate
PI3K: Phosphatidylinositol 3-kinase
PIM1: Proto-oncogene serine/threonine-protein kinase
PKB/AKT: Protein kinase B
PKM: Pyruvate kinase M
PNS: Panax notoginseng saponins
PR: Progesterone receptor
PRC2: Polycomb repressive complex 2
PTBP1: Polypyrimidine tract-binding protein 1
PTX: Paclitaxel
qRT-PCR: Quantitative real-time PCR
RAB11A: RAB11A member RAS oncogene family
RBPs: RNA binding proteins
RIP: Ribonucleoprotein immunoprecipitation
ROCs: Receiver operating characteristic curves
SETD7: SET domain containing 7 histone lysine methyltransferase
SNHG6: Small nucleolar RNA host gene 6
TAK1: TGF-β-activated kinase 1
TAZ: Transcriptional coactivator with a PDZ-binding domain
TGFβ1: Transforming growth factor beta 1
TNBC: Triple-negative breast cancer
TNM: Tumor, nodes, and metastases
UKL1: Unc-51-like kinase 1
UTR: Untranslated region
VASP: Vasodilator-stimulated phosphoprotein
VPS45: Vacuolar protein sorting 45 homolog
WIPF-1: WAS/WASL interacting protein family member 1
YAP: Regulators of yes-associated protein
ZEB1: Zinc finger E-box binding homeobox 1
